# Granulomatous Mastitis: An Initial Presentation of Undiagnosed Prolactinoma

**DOI:** 10.7759/cureus.65639

**Published:** 2024-07-29

**Authors:** Hussam Alkaissi, Emily J Kim, Navid Salahi, Samy I. McFarlane

**Affiliations:** 1 Endocrinology, Diabetes and Metabolism, National Institutes of Health (NIH), Bethesda, USA; 2 Internal Medicine, Kings County Hospital Center, Brooklyn, USA; 3 Pathology, State University of New York Downstate Medical Center, Brooklyn, USA; 4 Internal Medicine, State University of New York Downstate Medical Center, Brooklyn, USA

**Keywords:** pituitary, benign breast condition, granulomatous mastitis, prolactinoma, prolactin

## Abstract

Granulomatous lobular mastitis (GLM) is a rare, benign inflammatory disease of the breast that shares some physical diagnostic features with breast cancer. GLM has been rarely reported to be associated with prolactinoma. In this report, we present a case of undiagnosed prolactinoma in a 37-year-old woman with its initial presentation as GLM. We discuss the underlying pathophysiologic mechanisms for the development of GLM and the potential immunomodulatory role of prolactin in the development of GLM. We also highlight the need to assess for possible prolactinoma in GLM, which might go undiagnosed as in the case of our patient who did not seek medical attention for her amenorrhea, which is likely due to hyperprolactinemia that might also have other clinical implications on cardiovascular and bone health due to consequent estrogen deficiency.

## Introduction

Granulomatous lobular mastitis (GLM) is a rare, benign, inflammatory breast disease of unclear etiology with a clinical presentation that is similar to breast cancer and periductal lobular mastitis. It occurs mainly in younger females and in association with autoimmune conditions [1]. Prolactin (PRL), a peptide hormone secreted from the anterior pituitary gland, plays an important role in lactation and the development of mammary glands within breast tissues. In addition to its lactogenic effect, it has over 300 other physiologic pleiotropic effects, several of which are immunomodulatory, including the expression and secretion of proinflammatory cytokines from macrophages [2]. As such, GLM has been connected to the presence of an underlying prolactinoma, in addition to other comorbid conditions, mostly autoimmune and rheumatological. In this report, we describe a case of GLM in the setting of prolactinomas, with a literature review of GLM and the immunological role of PRL in pathogenesis.

This case report was presented as an abstract at the Endocrine Society Meeting in 2023, entitled "Granulomatous Mastitis: An Unusual Presentation of Prolactinoma."

## Case presentation

**Figure 1:**
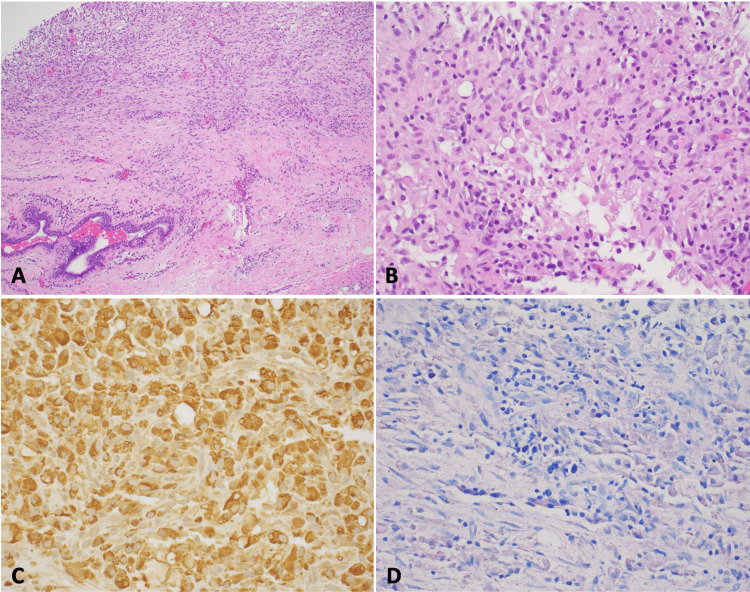
Poorly formed non-caseating granulomas (A. H&E, 100×, B. H&E, 400×). C. The CD68 highlighted histiocytes. D. No microorganisms are stained on the AFB stain H&E: hematoxylin and eosin; AFB: acid-fast bacilli

A 37-year-old woman presented with right breast pain and swelling for four weeks and amenorrhea for five years, for which she did not seek medical attention. Prior to that, she had no medical issues, her first menstrual period was at age 12, and until age 32, her periods were regular. She was gravida 1, para 1, with normal spontaneous vaginal delivery at the age of 20. She has no previous surgeries, no smoking or alcohol use history, and no family history of breast cancer, autoimmune, or rheumatological disorders. Physical examination showed non-bloody, non-purulent nipple discharge bilaterally, with small, firm, mildly tender retro-areolar mass in the right breast, with intact overlying skin, and no enlarged axillary lymph nodes. The rest of the exam was otherwise unremarkable. A breast ultrasound showed a 1 cm complex cystic mass, which prompted a biopsy that showed benign breast tissue with noncaseating granulomas formed by macrophages (Figures 1A-1B), positive for CD68 on immunohistochemistry (Figure 1C).

Ziehl-Neelsen staining for acid-fast bacilli was negative (Figure 1D). Polymerase chain reaction (PCR) for Mycobacterium tuberculosis, and interferon-gamma release assay (IGRA) were negative. Staining for fungi was also negative. Given the findings that are suggestive of granulomatous mastitis, with its known association with prolactinoma, and our patient's history of secondary amenorrhea, an additional workup revealed a prolactin level of 237 ng/mL (reference range: <25 ng/mL). Thyroid-stimulating hormone (TSH), insulin-like growth factor 1 (IGF-1), and adrenocorticotropic hormone (ACTH) were otherwise within normal range. Pituitary MRI showed a lesion with diminished enhancement measuring 2.8 x 3.2 x 4.2 mm within the left side of the pituitary gland, suggestive of a pituitary microadenoma, with no effect on optic chiasm, or cavernous invasion, given the small size of the adenoma. The patient was started on bromocriptine 2.5 mg daily, which resulted in the normalization of prolactin levels and the resolution of the right breast's lesion within three months. The menstrual period resumed six months after treatment.

## Discussion

Granulomatous lobular mastitis (GLM) was first described in a series of five patients in 1972 by Kessler and Wolloch as a cancer-simulating breast lesion. Since then, there have been advances in identifying clinical and histological characteristics of patients with GLM [1,3]. Proposed predisposing factors include nipple retraction, breastfeeding, multiple pregnancies, oral contraceptives, milk stasis, elevated prolactin, and trauma to the breast tissue [4]. Additionally, factors that increase prolactin levels have been associated with GLM, such as prolactinoma, and medications that block dopamine-2 receptors such as antipsychotics [5,6].

The most widely accepted pathogenesis model in GLM is an immune response involving both humoral and cell-mediated immunity induced by retained milk [7]. Lactation disorders or hyperprolactinemia can lead to secretion retention of milk in the breast duct and injury to the epithelial cell, leading to increased milk duct permeability. Both retention and permeability encourage secretion overflow into the surrounding lobular mesenchyme and cause local inflammation, with infiltration of T-cells and macrophages, causing delayed hypersensitivity (non-caseating granulomas) and B-cells, antibodies production, and additional systemic manifestation [7,8]. Such extramammary manifestations of GLM include arthritis/arthralgia and erythema nodosum, with positive responses to corticosteroid or methotrexate treatment [7]. While much of the focus in GLM pathogenesis is on the intraluminal secretion of milk and its stasis (regardless of prolactin level), prolactin itself may play a larger immunologic role in influencing the development of GLM. Such a prolactin-centered model is based on the prolactin immunologic profile and the fact that some patients do not fit the profile of patients with lactation disorders such as the previously described male patients and nulliparous patients [9,10]. The common characteristic among our cases and these other cases is the elevated prolactin level.

PRL has an immunomodulatory effect on several cells in the immune system, including B-cells, T-cells, and macrophages. A study showed that immune function was restored in hypophysectomized rats when prolactin was reintroduced. In addition to low levels, PRL excess can result in immunocompromise, which was demonstrated in lactating female and prolactin-treated male rats [11]. Prolactin has cytokine-like effects and regulates the expression of genes crucial to leukocyte function. When released into the immune system, PRL can control lymphocyte response by paracrine and autocrine mechanisms [12]. Because of this, prolactin abnormalities have been described in many autoimmune conditions such as autoimmune uveitis, thyroid disease, and systemic lupus erythematosus [11,12].

Additionally, PRL facilitates metalloproteinase activity that can degrade extracellular matrices at the feto-maternal interface, which could explain why hyperprolactinemia can cause recurrent miscarriages [13]. Along this train of thought, it is possible that excessive prolactin in the breast tissue could cause collagenolysis and further contribute to GLM.

In a recent study by Bi et al., a metagenomic examination of 25 samples of granulomatous mastitis identified unusual organisms in 68% of the samples. Of these, *Corynebacterium kroppenstedtii* was the most common (60%), followed by *Pseudomonas oleovorans* (16%), Epstein-Barr virus (4%), *Acinetobacter baumannii* (4%), and *Tepidiphilus thermophilus* (4%). In addition, 50% of the patients had elevated PRL and elevated immunological markers such as C3 and IgA [14]. This suggests that GM results from an interaction between the presence of specific bacteria that may otherwise be non-pathogenic, but in certain individuals, with an added elevation in prolactin and its effect on the immune system, may result in granulomatous lobular mastitis.

Clinically, several comorbid conditions were found in several larger case series and case reports of granulomatous mastitis. One of the most common associations is autoimmune diseases. In a study conducted by Martinez-Ramos et al., 70 published reports on granulomatous mastitis from different parts of the world showed that the country with the highest number of reports was Turkey, and autoimmune/rheumatological disorders were present in up to 34% of patients [15]. As such, the most common treatment modality was glucocorticoids, and surgery was a second line. Local pain in the breast lesion was present in 66% of the cases. Prolactin levels were not assessed in this study. Several conditions were reported in another bibliographic analysis focusing on autoimmune associations with GLM. Such associations included systemic lupus erythematosus (n=3), systemic sclerosis (n=1), rheumatoid arthritis (n=5), psoriasis (n=1), ankylosing spondylitis (n=2), familial Mediterranean fever (n=1), sarcoidosis (n=44), and granulomatosis with polyangiitis (n=15) [16]. There is a clear abundance of cases associated with granulomatous formation (i.e. sarcoidosis and granulomatosis with polyangiitis) with granulomatous mastitis. An overlooked association is one with psychiatric illnesses treated with anti-dopaminergic agents, given the associated adverse effect of these agents on prolactin levels. In a retrospective analysis of patients treated with antipsychotic agents, 19 cases of granulomatous mastitis were identified and several were treated by lowering the dose of antipsychotic medications, switching, or even discontinuation [17]. In a retrospective study by Huang and Wu, analyzing risk factors for the recurrence of granulomatous mastitis after treatment, higher body mass and index, and higher FSH/LH ratio were significantly associated with a higher risk of recurrence. Interestingly, prolactin levels tended to decrease after treatment, and in those whose prolactin levels did not decrease, the odds ratio (OR) of recurrence was 21.4, making prolactin level changes one of the highest associations and an independent risk factor for recurrence [18]. Risk recurrence can be as high as 24%, as reported in a large cohort of 474 patients by Azizi et al., with skin involvement being one of the common risk factors for recurrence [19]. In addition to higher BMI, higher FSH/LH ratio, lack of reduction of prolactin levels, and skin involvement, other risk factors for recurrence include smoking (OR=48.5) and infection with *Corynebacterium kroppenstedtii* (OR=32.2) [20].

## Conclusions

Although rare, granulomatous lobular mastitis is an inflammatory condition that, in addition to causing much pain and discomfort to the patients, GLM poses a diagnostic dilemma given its tumoral presentation that mimics the more grave diagnoses of breast cancer. We also highlighted the need for clinicians to be aware of the association between GLM and prolactinoma, which might be either asymptomatic or undiagnosed. Current management is multifold and includes supportive treatment, corticosteroids, immunosuppressive agents, and sometimes surgical drainage. Prolactin has endocrine and immunologic functions and may play a role in the pathogenesis of GLM. With increasing evidence of the various immunomodulatory effects prolactin has, it is worth further exploring, especially in relation to inflammatory lesions and conditions such as granulomatous lobular mastitis.
